# Editorial: Evidence on low-cost technologies for neurological rehabilitation in low and middle-income countries

**DOI:** 10.3389/fneur.2023.1323808

**Published:** 2023-12-13

**Authors:** Sureshkumar Kamalakannan, Bindu Menon, John M. Solomon, Kamarul Imran Musa

**Affiliations:** ^1^Social Work Education and Community Well-Being, Northumbria University, Newcastle upon Tyne, United Kingdom; ^2^Public Health Foundation of India, New Delhi, India; ^3^Apollo Speciality Hospitals, Nellore, Andhra Pradesh, India; ^4^Department of Physiotherapy, Manipal College of Health Professions, Manipal Academy of Higher Education, Manipal, Karnataka, India; ^5^Community Medicine, Universiti Sains Malaysia Health Campus, Kota Bharu, Kelantan, Malaysia

**Keywords:** digital technology (DT), neuro rehabilitation, low- and middle-income countries, low-cost, interventions-long term/chronic illness

Neurological Disorders constitute a significant burden in low- and middle-income countries (LMICs). Limited access to rehabilitation, paucity of evidence for neurological rehabilitation, and priority to preventive aspects in LMICs have been the neglected reasons for this burden (1). The application of technologies to address unmet needs has been found relevant. Therefore, the editors proposed to gain useful insights on this topic. Four articles were included to describe the status of this topic.

From the articles published on this topic, it is clear that the development of technological innovations for neurological rehabilitation in LMICs is rapidly emerging. The systematic approach to co-design, co-production, development, and evaluation is evident. The methods to generate and bridge the gaps in evidence on the needs and perspectives of caregivers as well as care providers were explored in-depth (Sidek, Kamalakannan et al.). Technological innovations for rehabilitation were considered important, and they primarily targeted the communities, particularly when people with neurological disabilities were discharged from hospitals to the community where neurological rehabilitation services were hardly available (2).

However, we could only indirectly understand the cost implications of these technological innovations. Given the diverse range of disabilities following neurological disability and inaccessibility to rehabilitation services in LMICs, cost is an important implication. Articles on this topic published by Sidek, Tengku Ismail et al. and Kamalakannan et al., talk about advanced technology optimizing virtual reality, and asynchronous and synchronous digital solutions. These innovations seem expensive, particularly in the context of LMICs where available rehabilitation services are not government-funded, and consumers must access them by spending from their pocket with significant opportunity cost. Hence there is a definitive need to consider the cost of not just developing a technological innovation but also the cost implications of integration and implementation of these technologies in LMICs (3).

Overall, the evidence on technologies for neurological rehabilitation is in its nascent stages in LMICs and it is aimed at bridging the gaps in access to services. The cost implications are yet to be explored in these contexts, but it provides a clear implication for high-income countries to consider the cost for aspects related to the implementation and integration of these digital solutions to be scalable. If economic evaluations on digital solutions could be achieved, the paucity of evidence for optimizing these scalable digital solutions for neuro-rehabilitation could be bridged worldwide.

## Author contributions

SK: Conceptualization, Resources, Supervision, Validation, Writing—original draft, Writing—review & editing. BM: Conceptualization, Supervision, Validation, Writing—original draft, Writing—review & editing. JS: Conceptualization, Supervision, Validation, Writing—original draft, Writing—review & editing. KM: Conceptualization, Supervision, Visualization, Writing—original draft, Writing—review & editing.
